# A genetic variant c.553G > T (rs2075291) in the apolipoprotein A5 gene is associated with altered triglycerides levels in coronary artery disease (CAD) patients with lipid lowering drug

**DOI:** 10.1186/s12872-018-0965-3

**Published:** 2019-01-03

**Authors:** Neda M. Bogari, Ashwag Aljohani, Amr A. Amin, Faisal A. Al-Allaf, Anas Dannoun, Mohiuddin M. Taher, Atalla Elsayed, Dareen ibrahim Rednah, Osama Elkhatee, Massimo Porqueddu, Francesco Alamanni, Soud Abdulraof A. Khogeer, Ahmed Fawzy

**Affiliations:** 1 Medical Genetics department, Faculty of Medicine, Umm Al-Qura University, Makkah, Kingdom of Saudi Arabia; 2 Biochemistry Department, Faculty of Medicine, Umm Al-Qura University, Makkah, Kingdom of Saudi Arabia; 3 Faculty of Medicine, AinShams University, Giza, Egypt; 4 Science and Technology Unit, Umm Al-Qura University, Makkah, Kingdom of Saudi Arabia; 5 ST JAMES’S HOSPITAL-Republic of Ireland, Ireland Dublin,; 6 Occupational Medicine, the University of Manchester, Manchester, UK; 7 Orthopedic residant, Fakeeh hospital, Jeddah, Kingdom of Saudi Arabia; 8 Department of Cardiology, Dalhousie University Halifax, Halifax, Nova Scotia Canada; 9 Department of Cardiac Surgery, King Fahd Armed Forces Hospital, Jeddah, Kingdom of Saudi Arabia; 10 Department of Cardiac Surgery, Head of Cardiac Surgery, Monzino Heart Center - University of Milan, Milan, Italy; 11 Division of Human Genetics and Genome Research, Department of Molecular Genetics and human Enzymology, National Research Centre, 33Bohouth St. Dokki, Giza, Egypt

**Keywords:** Triglyceride, APOA5 gene, Polymorphism, Genetic variation, Atorvastatin, Lipid lowering drugs, Coronary artery disease, Kingdom of Saudi Arabia(KSA)

## Abstract

**Background:**

Elevated plasma triglycerides (TGs) are widely used as a major cardiovascular risk predictor and are thought to play an important role in the progression of coronary heart disease (CHD). It has been demonstrated that lipid lowering was associated with lower mortality in patients with CHD. The present study therefore aimed to investigate the consequences of the genetic variant c.553G > T (rs2075291) in apolipoprotein A5 gene to determination of triglycerides levels in CAD patients receiving, atorvastatin, lipid lowering drug.

**Methods:**

We here report that a recently identified genetic variant, c.553G > T in the APOA5 gene which causes a substitution of a cysteine for a glycine residue at amino acid residue 185(G185C) is also associated with increased TG levels. To investigate theses effects, a case-control study compressing 608 subjects from the same area was performed.

**Results:**

TG levels in T allele patients were significantly lower than the control GT allele patient (χ^2^ = 2.382E2^a^, *P*-value < 0.001). Overall, patients carrying T allele showed lower levels of TG than patients carrying GG allele. The homozygous patient for the T allele presented normal cholesterol levels of 134 mg/dl, and the levels in GG patients ranged from 25 to 340 mg/dl (P-value < 0.001). In summary, we demonstrated that the presence of c.553G > T variant (rs2075291); in APOA5 gene increases human plasma TG levels.

**Conclusion:**

Nevertheless, T allele is found to reduce TG levels in CAD patients who are on the cholesterol medication, atorvastatin. Thus, c.553G > T variant can be considered as a significant predicator of hypertriglyceridemia. In addition, it could be used as a hallmark for the diagnosis and prognosis of CAD.

## Background

Coronary artery disease (CAD) is one of the most commonly diagnosed heart diseases. Diabetes, an elevation in blood pressure and high-fat diets are considered as some of the factors that contribute to the causation of CAD [1]. Among these factors, an elevation in triglycerides (TG) levels have shown a correlation with CAD occurrence although the exact mechanism is still obscure [2]. Hypertriglyceridemia can develop as a result of primary factors including mainly genetics, or secondary factors such as diabetes and hyperlipidemia [2]. Genetic abnormalities have contributed in causing hypertriglyceridemia through influencing the metabolism of triglycerides [3]. The overproductions of apo C-III through genetic modifications and Lipoprotein lipase (LPL) have shown an association with variations in triglycerides levels [4].

Recently, the sequencing of human genomic DNA has led to the discovery of Apolipoprotein A5 (APOA5) gene which belongs to a regulatory gene family including APOA1, APOC3 and APOA4 [5].This gene is located on chromosome 11q23 in about 30 kb downstream of APOA4, and contains four exons. Several studies have indicated that the newly identified apolipoprotein locus plays a major role in triglycerides hemostasis [6]. It encodes for apoA-V protein which reduces triglycerides plasma levels [6]. Therefore, genetic alterations in APOA5 could result in changes in TG levels. Point mutations in APOA5 yield incomplete assembly of apoA-V protein, and were observed mostly in patients with hypertriglyceridemia. Nevertheless, no specific mutations were known to cause severe illnesses, but certain studies have predicted the association of single nucleotide polymorphisms (SNPs) in APOA5 with medical conditions. The variants -1131 T > C and c.56C > G (S19 W) for APOA5s are examples of SNPs that have correlations with high triglycerides plasma levels [7].Accordingly, A novel variant c.553G > T was lately identified through the sequencing of APOA5 coding region. This SNP causes a substitution of the amino acid cysteine with a glycine molecule [4]. The effect of which has been researched and the conclusion is still to be reached. Upon the effort to study c.553G > T variant, a very recent research revealed that introducing a free cysteine in APOA-V protein allows the binding of it with other proteins through the formation of disulfide bonds which affects TG modulation [8]. Furthermore, c.553G > T variant (rs2075291); was detected in a higher rate in patients with acute coronary syndrome when compared to the control group [1]. Atorvastatin is a commonly known drug that is used to regulate lipid metabolism [9]. Based on Biopharmaceutics Classification System, atorvastatin is considered as a class II drug [10], and is a member of statins family also known as 3-hydroxy-3-methylglutaryl coenzyme A reductase inhibitors [11]. Therefore, atorvastatin is found to reduce cholesterol levels in CAD patients. In a study that was conducted to analyze the effects of atorvastatin on various lipoproteins, atorvastatin showed no effects on TG levels in Chylomicron, LDL, IDL and VLDL while it reduced cholesterol levels in these lipoproteins [12]. Here, we examine the effects of the genetic variant c.553G > T (rs2075291); on TG levels in CAD patients receiving atorvastatin daily as a lipid lowering medicine.

## Methods

### Human subjects

This study was approved by the Institutional Review Board and the Research ethics committees (REC) in Umm al-Qura University medical school at Makkah, Kingdom of Saudi Arabia. Blood was obtained from the subjects after obtaining informed written consent from the patients or their representatives. Patients with CAD confirmed by coronary angiography (> 50% stenosis in one or more arteries and stable or unstable angina pectoris) were enrolled in the study. Healthy controls from the same area were also included. Most of these patients were on appropriate and recommended doses of statin therapy as per guidelines for the management of coronary artery disease. The duration of therapy was not captured in current study.

All controls were examined clinically and investigated by electrocardiography to exclude clinically apparent CAD or other cardiovascular disease.

### Laboratory analysis

An amount of 5 ml blood samples were obtained from the subjects in the morning after an overnight fast. Serum lipid parameters, including triglyceride (TG), total cholesterol (TC), high-density lipoprotein cholesterol (HDL-C) and low-density lipoprotein cholesterol (LDL-C), apolipoprotein were measured by Dimension® Clinical Chemistry System using Flex reagents.

### DNA genotyping

**Fig. 1:**
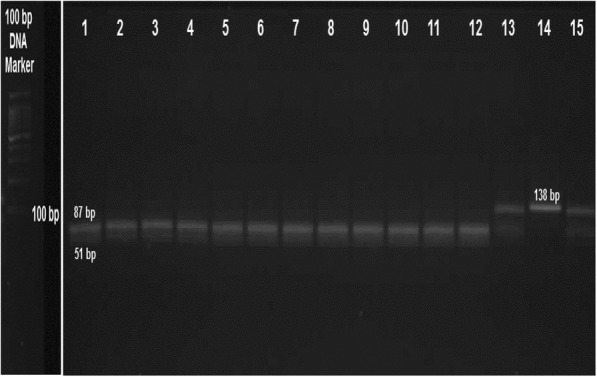
RFLP analysis result of *APOA5* c.553G > T locus. The products were separated on a 2% agarose gel and stained with ethidium bromide. Lanes from 1 to 12; are considered as GG homozygous (87 pb and 51 pb), Lanes; 13 and 14 are considered as GT heterozygose (138 pb, 87 pb and 51 pb), Lane 14 is considered as TT homozygose (138 pb)

Genomic DNA was extracted from peripheral blood samples using GeneJET Whole Blood Genomic DNA Purification kit (Thermo Scientific Co. Ltd.) and was stored at − 20 °C. Genotyping for c.553G > T (rs2075291) was performed by polymerase chain reaction (PCR) and restriction enzyme digestion. The primers were as follows: Forward: 5′-AGA CAC CAA GGC CCA GTT GCT GGG ‘3, Reverse: 5’-ATG CCG CTC ACC AGG CTC TCG GCG ‘3. The PCR amplification was performed in Veriti thermal cycler (Life technologies co.) DNA was amplified using AmpliTaq Gold 360 Master Mix (Life Technologies co.) under the following conditions: initial denaturation at 95 °C for 5 min, followed by 37 cycles of 95 °C for 30 s, 58 °C for 30 s, and 72 °C for 1 min, and a final extension step at 72 °C for 10 min. These primers yielded a PCR fragment of 138 bp. Subsequently, 5 U of the enzyme HaeIII (New England BioLabs Inc.) was added to 20 μl PCR products in a total volume of 25 μl and incubated at 37 °C over night. After restriction enzyme digestion, the products were separated on a 3% agarose gel and visualized using ethidium bromide and ultraviolet light. We found fragments of 138 and 87 bp for the GT heterozygotes, a single 138 bp product for the TT homozygotes, and a single 87 bp product for the GG homozygotes (Fig. 1).

## Results

### Baseline characteristics

Five hundred eighty five subjects including 247 females (130 of them were considered as controls while 117 were assigned as cases) and 338 males (107 of them were controls while 231 were cases) were evaluated for this study. The baseline characteristics of the CAD and control groups are shown in Table 1. There was no statistical significant difference in gender between the two groups. However, the results showed a perceptible difference regarding to patients’ age even though the control group was somewhat younger than CAD patients. Other parameters including blood pressure and glucose levels were found to be higher in the control group than CAD patients. Lipid parameters such as LDL and HDL levels did not show any significant differences between the two groups. Nevertheless, cholesterol and triglycerides levels were higher in CAD patients than control group even though some of them are on atorvastatin.

**Table 1:** Baseline characteristics of CAD and control groups

	CAD (354)	Control (377)	*P*-value
Age (years)	58.52 ± 11.10	37.3 ± 14.7	0.000
Gender (Male/ Female)	235/119	246/131	0.9009
Hypertension (n)	1.31 ± 0.51	1.78 ± 0.41	0.001
Glucose (mmol/ l)	1.39 ± 0.53	1.82 ± 0.38	0.000
HDL-C (mmol/ l)	1.37 ± 0.56	1.46 ± 0.63	0.103
LDL-C (mmol/ l)	1.65 ± 0.96	1.55 ± 0.80	0.191
Chol-C (mmol/ l)	1.17 ± 0.46	1.10 ± 0.36	0.041
TG-C (mmol/ l)	1.51 ± 0.82	1.16 ± 0.46	0.000

No statistical significant difference in gender between the two groups. However, the results showed a perceptible difference regarding to patients’ age even though the control group was somewhat younger than CAD patients. Other parameters including blood pressure and glucose levels were found to be higher in the control group than CAD patients. Lipid parameters such as LDL and HDL levels did not show any significant differences between the two groups. Nevertheless, cholesterol and triglycerides levels were higher in CAD patients than control group even though some of them are on atorvastatin.

### Relationship between T allele and triglycerides levels

TG levels in patients carrying the wildtype allele differ depending on whether they are on atorvastatin, a drug that lower cholesterol levels in the plasma, or not. CAD patients who were on atorvastatin presented higher TG levels than patients who were not on this drug (average TG levels for both groups were 143.5 mg/dl and 125.8 mg/dl respectively). Regarding to the T allele presence, two of the male patients have the T haplotype in APOA5 gene while only one patient has TT allele. One of the heterozygous GT allele patients and the TT allele patient are on atorvastatin. On the other hand, the other GT allele patient is not on atorvastatin and is considered as a control in this study. TG levels in T allele patients were significantly lower than the control GT allele patient (χ^2^ = 2.382E2^a^, *P*-value < 0.001). Overall, patients carrying T allele showed lower levels of TG than patients carrying GG allele. See Table 2.

**Table 2:** TG levels in CAD patients separated by APOA5 genotype. The wildtype represents GG allele while GT and TT alleles indicate the presence of c.553G > T

Parameter	ApoA5 genotypes			*P*-value
	GG	GT	TT	
TG (mg/dl)	35–533	170–277	71–96	0.001

### Association of T allele with other lipid parameters

**Fig. 2:**
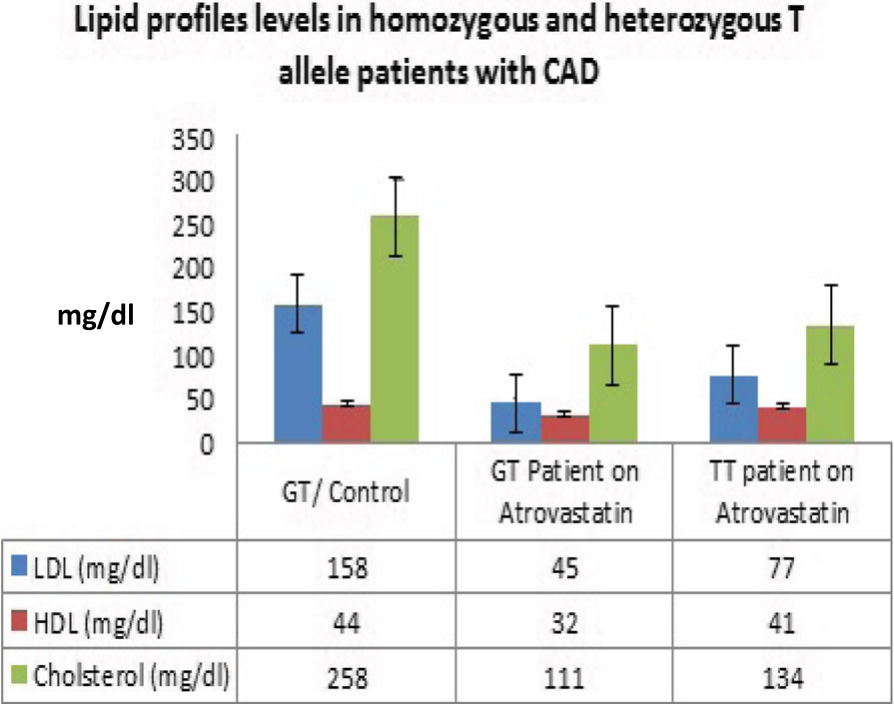
Lipid profiles levels in homozygous and heterozygous T allele patients with CAD separated by APOA5 genotype. The variants (GT and TT) are defined by the presence of c.553G > TSNP

In addition to TG levels, low density lipoprotein (LDL), High density lipoprotein (HDL) and cholesterol levels were measured. Results from the analysis showed minor differences between patients carrying the wildtype and minor T alleles. CAD patient carrying TT allele and GT allele patient on Atorvastatin have normal LDL levels (77 mg/dl and 45 mg/dl) respectively. On the other hand, CAD patient carrying GT allele but not on atorvastatin has slightly high yet normal LDL level (158 mg/dl). LDL levels vary in patients with the wildtype allele (11 to 199 mg/dl). There was no significant difference in HDL levels between all CAD patients carrying GT and TT alleles, and the range of which varies from 32 to 44 mg/dl. Finally, regarding to cholesterol levels in CAD patients with the minor T allele, the patient with GT allele on cholesterol medication has normal cholesterol level (111 mg/dl) while cholesterol level in the other GT patient was high (258 mg/dl). The homozygous patient for the T allele presented normal cholesterol levels of 134 mg/dl, and the levels in GG patients ranged from 25 to 264 mg/dl (*P*-value < 0.001). See Fig. 2.

### Association of T allele with other factors

**Fig. 3:**
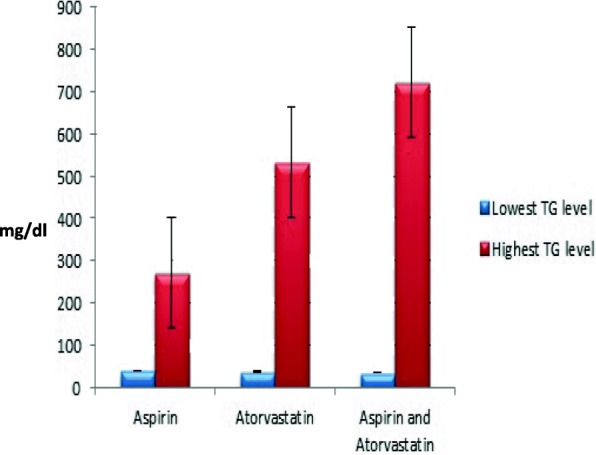
TG levels in homozygous G allele patients with CAD. Values are separated into three groups depending on the type of medications the patients are taking

**Fig. 4:**
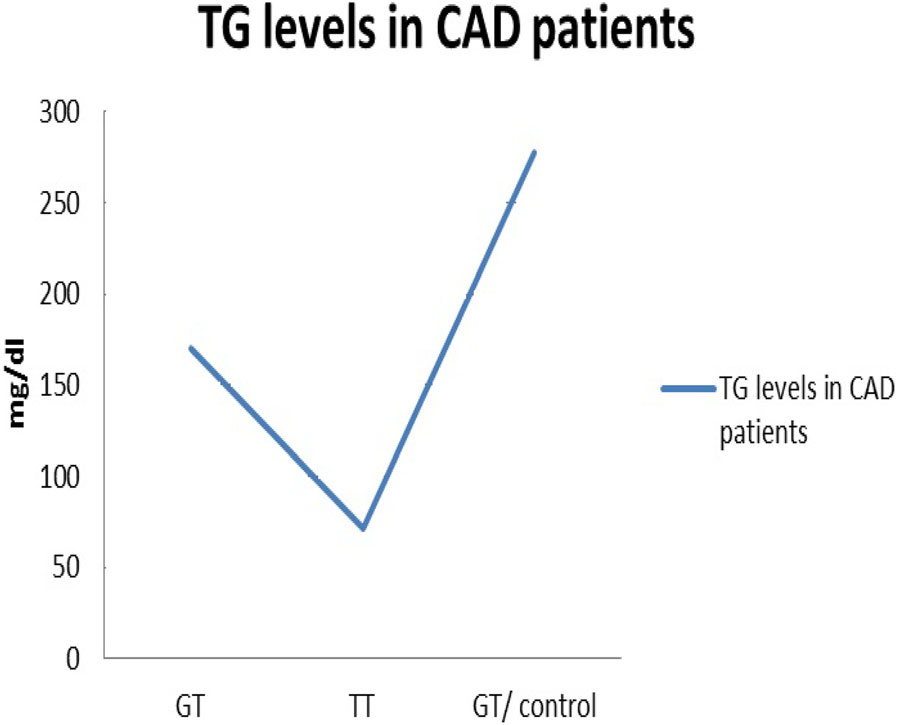
TG levels in CAD patients who have c.553G > T variant. GT/ control represent a patient with GT haplotype and is not taking atorvastatin or aspirin while GT and TT alleles carriers are taking both atorvastatin and aspirin

Other parameters such as smoking status, diabetes and exercising were included in this study. In T allele patients, the T allele homozygous patient is diabetic who smokes and does not exercise. Conversely, the patients who are heterozygous for the T allele were non-smokers, non-diabetic and performed exercises. The differences in previous parameters showed no significant effects on their TG levels. Therefore, these parameters seem to have no influence on the T allele presence. On the other hand, there was a difference in the outcomes of patients who were on aspirin. In T allele patients, the two patients who were taking Aspirin had lower TG levels of 71 and 170 mg/dl than the patient who was not taking aspirin, the control. See Table 1. In addition, patients with the wildtype allele and who were on aspirin and atorvastatin presented the lowest TG level of 35 mg/dl when compared to GG patients who were either on aspirin or atorvastatin with TG levels of 40 and 37 mg/dl, respectively. See Fig. 2 and Figs. 3, 4.

## Discussion

Our study validate the important role for CAD as the most frequent cause of mortality across the world according to data obtained from the World Health Organization (WHO) [13]. Causes of such unfortunate disease may be due to the complex interplay between genetic risk factors and environmental exposures that occur at critical times in development. Several epidemiological studies have shown that apolipoprotein A1/C3/A4/A5 gene cluster is found to be one of the factors that could cause premature CAD [14]. Among the gene cluster, APOA5 gene appears to affect TG levels [1]. Recently, several SNPs found in APOA5 cause an elevation in TG levels. A study has indicated that one of APOA5 SNPs, − 1131 T > C (rs2075291) variant, affects TG and HDL levels; therefore, increases the risk of developing cardiovascular diseases and diabetes [15]. Another study has shown that upon the transduction of AAV2/8-LacZ, AAV2/8-WT apoA-V and AAV2/8-G162C apoA-V, TG levels were lower in the WT mice when compared to both mutants [16]. In this study, we have described the relevance between the variant G162C (corresponding to c.553G > T) and TG levels in CAD patients who are on atorvastatin daily.

The incidence of diabetes in general population in Saudi Arabia is very high with up to 40% in some reports. It is possibly that the control group has many undiagnosed, untreated or borderline patients. On the other hand the CAD group most likely would be well treated patients and that is why there glucose levels are better.

Potential limitation of this study should be addressed. As the number of patients in each cohort is small to make conclusive results. Furthermore, there are differences between the control group and CAD group that may affect the results. Larger number of patients with TT allele is required to study in detail the effect of statin therapy on TG levels. Additionally, the duration and response to treatment was not studied in this report.

The frequency of c.553G > T allele in CAD patients was higher than normal subjects. Normally, the presence of T allele raises TG levels; yet, in patients carrying this allele and are on atorvastatin, it seems to lower TG levels. However, in a previous study, Ikejiri et al., stated that atorvastatin has no reduction effects on TG rich lipoproteins [12]. That conclusion is true regarding to the findings of TG levels in GG allele patients which showed no direct effects of atorvastatin on TG levels. In addition, there were no substantial differences in LDL between patients carrying T allele or the wildtype allele. Recent studies have revealed that APOA5 could affect cholesterol homeostasis and could cause hypertriglyceridemia [17]. Recent studies have revealed that APOA5 could affect cholesterol homeostasis and could cause hypertriglyceridemia [17] it is possible that statin therapy in these patients have more effect on TG metabolism given its effect on cholesterol hemostasis. Further mechanistic studies needs to be done on these patients. Another study that included Chinese subjects has shown that in CAD patients and control groups, the wild type GG carriers have considerably lower TG levels in comparison to the T allele carriers [18]. Regarding to HDL levels in the study subjects, T allele patients who were on atorvastatin have lower HDL levels than the other T allele patient who was not taking atorvastatin, but all results were in the normal range. This has been proven earlier by another Chinese study that showed no significant associations between the presence of T allele and lipid parameters including LDL, TC, and HDL [19]. As expected, cholesterol levels in T allele patients on atorvastatin were normal and lower than the T allele patient who was not on atorvastatin. This result is due to the fact that the pharmacological effect of atorvastatin is to lower cholesterol levels in the plasma. Previous studies have confirmed that atorvastatin reduces both LDL and total cholesterol, and it decreases cholesterol levels in females more than males [20]. The clinical implications for TG and CAD is less well studied compared to LDL levels. There is some associations but not as important as LDL and cholesterol levels on CAD. Medications that lower TG such as fenofibrates failed to improve outcomes compared to statin therapy in patient with CAD. Thus fenofibrates is indicated only in patients with very high TG levels despite diet control.

Finally, both T allele and the wildtype allele patients who were taking atorvastatin along with aspirin showed the lowest TG levels when compared to patients who were not on aspirin and/ or atorvastatin. These findings suggest that aspirin has an effect on TG levels when is taking with cholesterol medications; yet, further studies are needed to determine if there is an effect depending on the presence or absence of T allele. A study that was done on mice has revealed that aspirin decreases the secretion of VLDL- TG from the liver which in turn reduces HFD- induced hypertriglyceridemia supporting our findings in this study [21].

## Conclusion

In summary, we demonstrated that the presence of c.553G > T (rs2075291) variant in APOA5 gene increases human plasma TG levels. Nevertheless, T allele is found to reduce TG levels in CAD patients who are on the cholesterol medication, atorvastatin. Thus, c.553G > T variant can be considered as a significant predicator of hypertriglyceridemia. In addition, it could be used as a hallmark for the diagnosis and prognosis of CAD.

## Abbreviations

APOA5: The apolipoprotein A5 gene
CAD: A coronary artery disease
SNP: Single nucleotide polymorphism
TG: Triglycerides

## References

[1] Ding Y (2012). Associations of polymorphisms in the apolipoprotein APOA1-C3-A5 gene cluster with acute coronary syndrome. J Biomed Biotechnol.

[2] Gotto AM (1998). Triglyceride as a risk factor for coronary artery disease. Am J Cardiol.

[3] Rade N, Pejic M, Daniel M, Lee T (2006). Hypertriglyceridemia. J Am Board Fam Med.

[4] Kao JT (2003). A novel genetic variant in the apolipoprotein A5 gene is associated with hypertriglyceridemia. Hum Mol Genet.

[5] Charlton-Menys V, Durrington PN (2005). Apolipoprotein A5 and hypertriglyceridemia. Clin Chem.

[6] Pennacchio LA, Rubin EM (2003). Apolipoprotein A5, a newly identified gene that affects plasma triglyceride levels in humans and mice. Arterioscler Thromb Vasc Biol.

[7] Vaessen SF (2006). Apolipoprotein A-V, triglycerides and risk of coronary artery disease: the prospective epic-Norfolk population study. J Lipid Res.

[8] Sharma V (2014). Aberrant hetero-disulfide bond formation by the hypertriglyceridemia-associated p.Gly185Cys APOA5 variant (rs2075291). Arterioscler Thromb Vasc Biol.

[9] Jing F (2016). Effects of atorvastatin combined with low-molecular-weight heparin on plasma inflammatory cytokine level and pulmonary pathophysiology of rats with sepsis. Exp Ther Med.

[10] Affandi MM, Tripathy M, Majeed AB (2016). Conductometric and volumetric studies of atorvastatin in aqueous solution of arginine from 298.15 to 313.15 K. J Adv Pharm Technol Res.

[11] Shah RV, Goldfine AB (2012). Statins and risk of new-onset diabetes mellitus. Circulation.

[12] Ikejiri A (2004). Effects of atorvastatin on triglyceride-rich lipoproteins, low-density lipoprotein subclass, and C-reactive protein in hemodialysis patients. Metabolism.

[13] Bampali K (2014). Genetics and coronary artery disease: present and future. Hell J Cardiol.

[14] Qi L (2007). Associations of the apolipoprotein A1/C3/A4/A5 gene cluster with triglyceride and HDL cholesterol levels in women with type 2 diabetes. Atherosclerosis.

[15] Song KH (2013). Association of apolipoprotein A5 gene 1131TC polymorphism with the risk of metabolic syndrome in Korean subjects. Biomed Res Int.

[16] Sharma V (2014). Hypertriglyceridemia associated with the c.553G>T APOA5 SNP results from aberrant hetero-disulfide bond formation. Arterioscler Thromb Vasc Biol.

[17] Chien KL (2008). Genetic association study of APOA1/C3/A4/A5 gene cluster and haplotypes on triglyceride and HDL cholesterol in a community-based population. Clin Chim Acta.

[18] Yin RX, Li YY, Lai CQ (2011). Apolipoprotein A1/C3/A5 haplotypes and serum lipid levels. Lipids Health Dis.

[19] Tang Y (2006). A genetic variant c.553G > T in the apolipoprotein A5 gene is associated with an increased risk of coronary artery disease and altered triglyceride levels in a Chinese population. Atherosclerosis.

[20] Adams SP, Tsang M, Wright JM (2015). Lipid-lowering efficacy of atorvastatin. Cochrane Database Syst Rev.

[21] van Diepen JA (2011). Aspirin reduces hypertriglyceridemia by lowering VLDL-triglyceride production in mice fed a high-fat diet. Am J Physiol Endocrinol Metab.

